# M2 macrophage-derived lncRNA NORAD in EVs promotes NSCLC progression via *miR-520g-3p*/*SMIM22*/*GALE* axis

**DOI:** 10.1038/s41698-024-00675-x

**Published:** 2024-08-30

**Authors:** Qingtao Zhao, Bin Li, Xiaopeng Zhang, Huanfen Zhao, Wenfei Xue, Zheng Yuan, Shun Xu, Guochen Duan

**Affiliations:** 1 Department of Thoracic Surgery, Hebei General Hospital, Shijiazhuang, Hebei Province China; 2 Hebei Bio-High Technology Development Co.Ltd, Shijiazhuang, Hebei Province China; 3 Department of Pathology, Hebei General Hospital, Shijiazhuang, Hebei Province China; 4 Department of Nursing, Hebei General Hospital, Shijiazhuang, Hebei Province China; 5 Department of Thoracic Surgery, The First Hospital of China Medical University, Shenyang, Liaoning China

**Keywords:** Non-small-cell lung cancer, Cell death

## Abstract

Non-small cell lung cancer (NSCLC) constitutes the majority of lung cancer cases, accounting for over 80%. RNAs in EVs play a pivotal role in various biological and pathological processes mediated by extracellular vesicle (EV). Long non-coding RNAs (lncRNAs) are widely associated with cancer-related functions, including cell proliferation, migration, invasion, and drug resistance. Tumor-associated macrophages are recognized as pivotal contributors to tumorigenesis. Given these insights, this study aims to uncover the impact of lncRNA NORAD in EVs derived from M2 macrophages in NSCLC cell lines and xenograft mouse models of NSCLC. EVs were meticulously isolated and verified based on their morphology and specific biomarkers. The interaction between lncRNA *NORAD* and *SMIM22* was investigated using immunoprecipitation. The influence of *SMIM22*/*GALE* or lncRNA NORAD in EVs on glycolysis was assessed in NSCLC cell lines. Additionally, we evaluated the effects of M2 macrophage-derived lncRNA NORAD in EVs on cell proliferation and apoptosis through colony formation and flow cytometry assays. Furthermore, the impact of M2 macrophage-derived lncRNA NORAD in EVs on tumor growth was confirmed using xenograft tumor animal models. The results underscored the potential role of M2 macrophage-derived lncRNA NORAD in EVs in NSCLC. *SMIM22*/*GALE* promoted glycolysis and the proliferation of NSCLC cells. Furthermore, lncRNA NORAD in EVs targeted *SMIM22* and *miR-520g-3p* in NSCLC cells. Notably, lncRNA NORAD in EVs promoted the proliferation of NSCLC cells and facilitated NSCLC tumor growth through the *miR-520g-3p* axis. In conclusion, M2 macrophage-derived lncRNA NORAD in EVs promotes NSCLC progression through the *miR-520g-3p*/*SMIM22*/*GALE* axis.

## Introduction

Lung cancer (LC) stands as the most prevalent malignancy and a leading cause of cancer-related fatalities worldwide^1^. Non-small cell lung cancer (NSCLC) accounts for more than 80% of all LC cases, with many patients receiving a delayed diagnosis due to the late onset of clinical symptoms and shortcomings in screening methods. Despite significant advances in surgical and medical treatments, the 5-year survival rate for patients with LC remains disappointingly low^2^. Consequently, there is an urgent need for new and highly sensitive biomarkers for early NSCLC detection and target molecules for the development of innovative therapies.

Continuous interactions between cancerous cells and their local and distant environments are crucial for effective cancer progression and systemic spread. Exosomes, small extracellular vesicles (EVs) measuring 30–100 nm in size, are released into the extracellular space in various cell types, occurring in both normal and pathological conditions. These extracellular vesicles contain a variety of molecules, including proteins, RNA, and lipids, which impact the physiological and pathological states of host cells^3–6^. In addition to proteins, various types of nucleic acids, such as mRNAs, microRNAs, and other non-coding RNAs, have recently been identified in EVs, participating in various biological processes^6–8^. RNAs in EVs have been linked to numerous biological processes facilitated by EVs. These RNAs in EVs can be internalized by nearby or distant cells as EVs circulate, subsequently influencing the activities of recipient cells^9–11^. Their involvement in the transfer of genetic information among cancer cells has generated growing interest in the field of EVs.

Long non-coding RNAs (lncRNAs), characterized as non-coding RNAs exceeding 200 nucleotides in length, perform functions in a wide range of cellular processes, including the regulation of the cell cycle, apoptosis, and genome stability^12–15^. The functional role of lncRNAs is intricately linked to their specific subcellular location. LncRNAs located in the nucleus participate in gene regulation at epigenetic and transcriptional levels, affecting processes such as histone modifications^7,8^, regulation of DNA methylation^9^, chromatin remodeling^10^, as well as interactions with chromatin modification complexes^11^, transcription factors^12^, and nuclear proteins^13^. Meanwhile, those residing in the cytoplasm play important roles in post-transcriptional and translational gene regulation, interacting with cytoplasmic proteins^14^, modulating mRNA metabolism^15,16^, and acting as endogenous competitive RNAs (ceRNAs) that interact with microRNAs^17–19^. Consequently, lncRNAs are extensively involved in the proliferation, migration, invasion, and drug resistance of cancer cells^20–22^. These findings emphasize the potential importance of lncRNAs in NSCLC development and their potential as innovative markers for diagnosis and treatment. LncRNA non-coding RNA activated by DNA damage (*NORAD*) is a lncRNA located on Chr20q11.23 and has been identified as an oncogene in many carcinomas, which can promote cancer cell proliferation and metastasis^23^. It has been reported that lncRNA *NORAD* could act in the ceRNA network with some microRNA or by sponging microRNAs^24,25^. MiRNA-520g, a member of the miRNA-515 family, is located on the chromosome 19^26^. It was discovered that miRNA-520g induced drug resistance in colorectal cancer, associated with prognostic factors in breast cancer, and promoted ovarian cancer development^27,28^. Small integral membrane protein 22 (SMIM22, Gene ID: ENSG00000267795) is a newly discovered gene associated with cancer development^29,30^. UDP-galactose-4-epimerase (GALE) encodes UDP-galactose-4-epimerase and acts on protein and lipid glycosylation in cells. It facilitates two separate yet similar reactions: transforming UDP-glucose into UDP-galactose, and converting UDP-N-acetylglucosamine into UDP-N-acetylgalactosamineEVs^31^. It was revealed that overexpression of GALE is associated with differentiation grade of gastric cancer^32^. However, the role of SMIM22 or GALE in NSCLC have not been investigated yet. Also, the interaction between lncRNA *NORAD* and *miR-520g-3p*, SMIM22 or GALE in NSCLC was not studied previously.

Tumor-associated macrophages (TAMs) have gained recognition as crucial contributors to the progression of various tumors^33–35^. TAMs are typically categorized into two phenotypes: (I) classically activated macrophages (M1), which exhibit pro-inflammatory properties, and (II) alternatively activated macrophages (M2), which display anti-inflammatory characteristics^36,37^. Throughout cancer progression, TAMs may infiltrate tumors, reinforcing tumor growth, invasion, metastasis, and angiogenesis^38–40^. Additionally, TAMs can impede immune responses, induce immune tolerance, and reduce responses to conventional therapeutic approaches^38,41–43^. As a result, TAMs may participate in various mechanisms contributing to the advancement of NSCLC.

In this study, we have explored the potential role of M2 macrophage-derived EVs in NSCLC cells. Our research reveals that lncRNA NORAD in EVs in EVs targeted *SMIM22* and *miR-520g-3p* in NSCLC cells. Moreover, *SMIM22*/*GALE* promoted glycolysis and proliferation of NSCLC cells. Notably, lncRNA NORAD in EVs in EVs promoted NSCLC cell proliferation, as well as NSCLC tumor growth through the *miR-520g-3p* axis. These findings highlight the potential involvement of M2 macrophage-derived lncRNA NORAD in EVs in NSCLC progression.

## Methods

### Cell culture

The LL2, H1299, H1560, and A549 cell lines were procured from ATCC (Manassas, VA, USA) and cultured in T-75 flasks utilizing DMEM medium (Sigma, St. Louis, MO, USA) Supplementaryed with 10% EVs-depleted fetal bovine serum (FBS; Beyotime, Shanghai, China). The THP-1 cell lines, also sourced from ATCC, were cultured in T-75 flasks using 1640 medium (Hyclone, Seattle, WA, USA) enriched with 10% EVs-depleted FBS (Beyotime, China). The cells were maintained at 37 °C in a 5% CO_2_ atmosphere, with media changed every 48 h.

In specific experiments, THP-1 cells were subjected to phorbol 12-myristate 13-acetate (PMA) exposure for 72 h to generate M0 macrophages. Subsequently, M0 macrophages were treated with lipopolysaccharides (100 ng/mL) and IFN-γ (20 ng/mL) for 48 h to induce polarization into M1 macrophages. Alternatively, M0 macrophages were treated with IL-4 (20 ng/mL) and IL-13 (20 ng/mL) for 48 h to induce polarization into M2 macrophages.

### Clinical sample collection

From August 2022 to August 2023, a total of 31 paired NSCLC and adjacent non-tumor tissue (at least 3 cm from NSCLC tissue) samples were gathered at Hebei General Hospital. These patients had not undergone any anti-tumor treatments such as chemotherapy or radiotherapy prior to the specimen collection and surgical resection was considered as the best treatment. All patients were pathologically diagnosed and the tissues were collected during surgery then immediately frozen and stored at −80 °C. All patients provided informed consent, and the study received approval and oversight from the Ethics Committee of Hebei General Hospital, following the principles of the Declaration of Helsinki. The levels of LncRNA NORAD, SMIM22 (both mRNA and protein levels), and miR-520g-3p were assessed in NSCLC tissue and adjacent tissues, and their correlation was analyzed. The Receiver Operating Characteristic (ROC) curve for lncRNA NORAD, miR-520g-3p, or SMIM22 was examined in NSCLC patients. The clinicopathological factors of patients are listed in Supplementary Table 1. The study was reviewed and approved by the Medical Ethics Committee of Hebei General Hospital in accordance with the Declaration of Helsinki. All included patients gave their informed consent to participate. Consent for publication was obtained from all participants.

### Xenograft tumor models

We obtained 60 syngeneic C57BL/6 immunocompetent mice (half male, half female), aged 4 weeks, with body weight ranging from 21 to 25 g. These mice were maintained under specific pathogen-free conditions following NIH guidelines, with *ad libitum* access to sterile rodent chow and water. They were housed in sterilized filter-top cages and kept on a 12-h light-dark cycle. The mice were randomly allocated into 12 groups, each containing five mice. Subcutaneous injections of the mouse Lewis lung carcinoma (LLC) cell line LL2 cells (2 × 10^6^ cells per mouse) were administered, with tumor size and volume monitored every three days. Tumor volume was calculated using the formula: V(cm^3^) = 1/2 × length × width^2^. The study received approval and oversight from the Ethics Committee of Hebei General Hospital.

### EVs isolation and detection

EVs were collected using an ultrafiltration and ultracentrifugation-based method. Briefly, culture medium Supplemented with 10% EVs-depleted FBS was collected and centrifuged at 800 × *g* for 5 min and additional 2000 × *g* for 10 min to remove lifted cells. The supernatant was subjected to filtration on a 0.1 µm pore polyethersulfone membrane filter to remove cell debris and large vesicles, followed by concentration by a 100,000 Mw cut-off membrane. The volume of supernatant was reduced from approximately 250–500 mL to approximately 30 mL. The supernatant was then ultracentrifuged at 100,000 × *g* for 1 h at 4 °C. The resulting pellets were resuspended in 6 mL PBS and ultracentrifuged at 100,000 × *g* for 1 h at 4 °C. The morphology of EVs was examined through transmission electron microscopy (TEM), and their size distribution was assessed using nanoparticle tracking analyser (NTA). The pelleted EVs were mixed with equal quantities of freshly prepared 2% glutaraldehyde in PBS, incubated overnight at 4 °C, postfixed with 1% osmium tetroxide in PBS at 4 °C for 2 h, and dehydrated in a graded series of ethanol. Following dehydration, the samples were transferred to propylene oxide and embedded in epoxy resin Quetol 812. Ultrathin sections were cut, stained with uranyl acetate and lead citrate, and observed. In brief, EVs were mixed and incubated with mouse anti-human CD63 monoclonal antibody for 1 h at room temperature. The sample was dropped onto the membrane surface of a copper mesh and incubated for 1 h at room temperature. After wash with PBS, immunogold conjugated goat anti-mouse IgG was dropped onto the membrane. The copper mesh was floated in the droplets with its membrane surface faced down at room temperature for 30 min. After wash with PBS, uranyl acetate drops were put onto the copper mesh surface and stained at room temperature for 30 s. EVs with black colloidal gold particles on the capsular membranes were marked as positive under the transmission electron microscope. Confirmation of EV markers was performed via western blotting.

NTA (Particle Metrix, Meerbusch, Germany) was used to determine the size of EVs. Firstly, the EV sample was diluted in pre-filtered PBS to ensure accurate measurements within the range of the NTA software (ZetaView 8.05.04). The software parameters included a temperature of 25 °C, sensitivity of 30–85 frames per second (fps), and a shutter speed of 55. Prior to analysis, the calibration of the instrument was performed using polystyrene particles from Microtrac GmbH with an average size of 100 nm. The data obtained from the analysis were processed using the NTA software (ZetaView 8.05.04).

### Glycolysis stress test

The extracellular acidification rate (ECAR) was measured using the Seahorse XF96 Extracellular Flux Analyzer (Seahorse Bioscience). ECAR is caused by lactic acid secretion in the extracellular space and reflects the glycolysis rate. Firstly, we measured glycolysis (baseline) with ECAR, which indicating the basal glycolysis rate to generate ATP for homeostatic maintenance of cells. Afterwards, we measured glycolytic capacity with ECAR, a measure of the maximum rate of conversion of glucose to pyruvate or lactate. A total of ~5 × 10^4^ cells/well were plated in XF24 microplates and received the corresponding treatment. On the following day, overnight-rested cells were resuspended in DMEM-based XF media (Seahorse XF base media Agilent, Santa Clara, CA, USA). After 60-min incubation in a non-buffered assay medium without CO_2_, the XF Cell Glycolysis Test Kit (Agilent, Santa Clara, CA, USA) was employed. The recording began with establishing baseline values, followed by sequential injections of glucose (10 mM), oligomycin (1 μM), or 2-DG (50 mM). For the determination of glycolytic capacity, cells in ECAR medium were incubated for 60 min at 37 °C in the presence of 10 mM of glucose or 1 μM oligomycin.

### Glucose uptake and lactate production assay

Glucose uptake was assessed by quantifying the glucose concentration in the medium (Sigma, St. Louis, MO, USA), and lactate production was evaluated in accordance with the manufacturer’s instructions using a Lactate Assay Kit (Solarbio, Beijing, China). Prior to the assessments, all metabolic parameters were standardized based on the total cell count.

### Immunoprecipitation (IP)

A549 cells underwent IP experiments. RNA-protein complexes were immunoprecipitated using a 3xFLAG antibody (Sigma, St. Louis, MO, USA). The fraction separated by IP was analysed via western blotting.

### Cell transfection

A549 cells were plated in 6-well plates at a density of 2 × 10^5^ cells/well and transfected with siRNA-lncRNA *NORAD*, *miR-520g-3p* mimic, or *miR-520g-3p* inhibitor using the Lipofectamine® 3000 kit (Invitrogen, Waltham, MA, USA). All siRNAs, miRNA mimics, and inhibitors were synthesized by GenePharma (Shanghai, China). Stable A549 cell lines with silenced lncRNA *NORAD*, over-expressed *miR-520g-3p*, or silenced *miR-520g-3p* were established through lentiviral transduction using the pCDH plasmid (System Biosciences).

### Cell viability analysis

Cell viability was assessed by the CCK-8 assay (Abcam, Cambridge, UK). A549 cells were initially plated in 96-well dishes at a concentration of 6 × 10^3^ cells/well and allowed to incubate for one day prior to any treatment. Following a 3 h incubation with CCK-8 solution, absorbance at 450 nm was determined using a microplate reader (PerkinElmer, Waltham, MA, USA).

### Colony formation assay

Cells were cultivated in 6-well dishes at a seeding density of 1 × 10^3^ cells/ well, and the culture medium was refreshed every 48 h. Following a 10-day incubation, the colonies were fixed and subsequently treated with crystal violet. The number of colonies was quantified.

### Cell apoptosis analysis

Transfected cells were enzymatically dissociated, and stained with an Annexin V apoptosis detection kit (Abcam) for apoptosis analysis. The rate of cell apoptosis was quantified through flow cytometry method. Approximately 1 × 10^6^ cells were collected, centrifuged at 500 g for 10 min, and washed twice with pre-cooled PBS. The supernatant was then discarded. Next, 500 μL of apoptosis-positive control solution was added and incubated on ice for 30 min. The cells were centrifuged again at 500 g for 10 min and washed with pre-cooled PBS. Pre-cooled 1 × Binding Buffer was added and mixed with the cells to bring the volume up to 1.5 mL. On the flow cytometer (BD, CantoII), Annexin V-FITC (excitation = 488 nm; emission = 530 nm) was detected through the FITC detection channel, and PI was detected through the PI detection channel (excitation = 488 nm; emission = 615 nm) using BD FACSDiva.8 software to collect 30,000 events. Data analysis was performed using flowJO 10.8. The cells were gated based on the physical parameters of FSC-A/SSC-A to identify the main cell population, and aggregates were removed using FSC-H/FSC-A. Finally, the proportion and number of apoptotic cells were analyzed using AnnexinV-FITC-A/PI-PE-A.

### EdU analysis

An EdU detection kit (Beyotime) was employed to evaluate cell proliferation. Cells were seeded in 96-well plates at a density of 5000 cells/well. Then, EdU labeling media were introduced to the 96-well plates at 37 °C for 2 h. After fixation with 4% paraformaldehyde, the cells were stained using the anti-EdU working solution and Hoechst 33342. Subsequently, a fluorescence microscope (Zeiss, Oberkochen, Germany) was used to visualize the cells.

### Dual-luciferase reporter assay

The 3ʹUTR of *SMIM22* mRNA, both the wild type (WT) and a mutated version (MUT) targeting the presumed *miR-520g-3p* binding site, were cloned into the luciferase reporter vector psiCHECK-2 (Promega, Madison, WI, USA). In luciferase reporter experiments, ~10,000 cells/ well were seeded in 24-well plates and transfected with the specified luciferase reporter vectors, along with *miR-520g-3p* mimics, *miR-NC*, *miR-520g-3p* inhibitor, or inhibitor NC, using the Lipofectamine® 3000 (Invitrogen). The relative luciferase activity within the cells was subsequently measured 48 h after transfection using the Dual-Luciferase Reporter Assay System (Promega).

### Fluorescence in situ hybridization (FISH) assay

The localization of lncRNA NORAD and miR-520g-3p in A549 cells was detected using the FISH assay with a FISH Kit (RiboBio, Guangzhou, China), following the manufacturer’s instructions. A549 cells on slices were fixed with 4% paraformaldehyde, then hybridized with a FAM-labeled probe for lncRNA NORAD and CY3-labeled probes for miR-520g-3p overnight at 37 °C. The slides were then counterstained with DAPI and visualized using a fluorescence microscope from Zeiss (Oberkochen, Germany).

### Gene expression profiling interactive analysis (GEPIA)

The Cancer Genome Atlas data analysis was conducted using the GEPIA website, as previously described^44^.

### Western blot analysis

Total protein was extracted from cultured cells utilizing RIPA buffer (Beyotime). Protein quantity and concentration were determined through a BCA assay (Beyotime). Subsequently, following suitable dilution, samples were loaded onto a 12% gel (Bio-Rad, Hercules, CA, USA), subjected to electrophoresis, transferred onto a 0.45 μm PVDF membrane (GE Healthcare, Chicago, IL, USA), and then blocked with non-fat dry milk (Beyotime). Primary antibodies were incubated overnight at 4 °C, followed by secondary antibodies for 2 h at 25 °C. The imaging process was carried out using a ChemiDoc MP Imaging System (Bio-Rad) with a chemiluminescent HRP substrate (Millipore, Billerica, MA, USA). GAPDH protein was employed as a reference control. The antibodies are listed in Supplementary Table 2.

### Immunofluorescence

Several cell slides were used for immunofluorescence. QuickBlock^TM^ immunostaining blocking reagent (Beyotime) was used to block non-specific binding sites, and 3% hydrogen peroxide was used to block endogenous peroxidase. Cells were fixed in 4% paraformaldehyde and permeabilized with 0.5% Triton X-100 in PBS at 25 °C for 15 min. Then, the samples were incubated with the primary antibody (rabbit monoclonal anti-SMIM22 (1:500), rabbit monoclonal anti-GALE (1:500)) at 4 °C overnight. After washing to remove the primary antibodies, the slides were incubated with secondary antibody at room temperature for 1 h and subsequently stained with 4′,6-diamidino-2-phenylindole (DAPI) (Keygen, China) for 5 min. Subsequently, a fluorescence microscope (Zeiss, Oberkochen, Germany) was used to visualize target proteins. EVs were labeled with a Green Fluorescent Kit against PKH67 (Sigma, St. Louis, MO, USA) following the manufacturer’s protocol.

### qRT-PCR

RNA extraction from cells or samples was achieved using Trizol reagent (Life Technologies, Carlsbad, CA, USA). After RNA isolation, cDNA synthesis was executed following the manufacturer’s guidelines with the cDNA Synthesis Kit (Vazyme, Nanjing, China). Quantitative PCR analysis was conducted using the SsoFast EvaGreen Supermix system (Bio-Rad). All expression data were normalized to GAPDH. The primers used in this study can be found in Supplementary Table 3.

### Statistical analysis

Statistical analysis was performed using SPSS v17.0 software (SPSS, San Diego, CA, USA). Quantitative data were subjected to one-way ANOVA, followed by multiple comparison Dunnett post hoc tests to determine statistical significance. Results are presented as mean ± standard deviation, with a significance level set at *p* < 0.05.

## Results

### Co-culturing with macrophages promoted proliferation of NSCLC cells

**Fig. 1:**
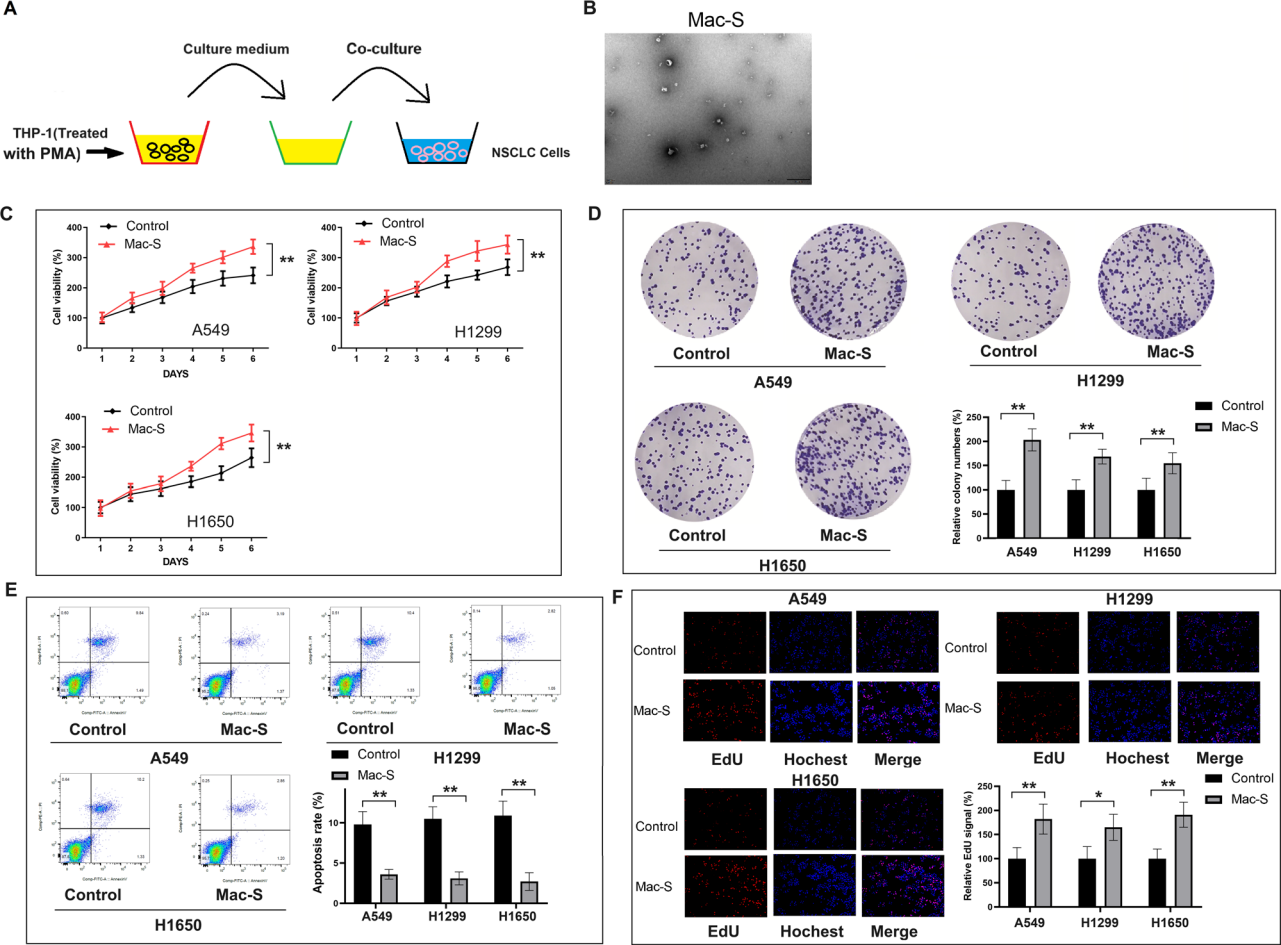
Co-culturing with macrophages promotes proliferation of NSCLC cells. **A** The strategy of co-culturing THP-1 cells and NSCLC cells. **B** Exosomes derived from THP-1 cells are identified by TEM and NTA. **C** The cell proliferation of NSCLC cells (including A549, H1299, and H1650 cells) after treatment with macrophage supernatant. **D** The colony formation of NSCLC cells (including A549, H1299 and H1650 cells) after treatment with macrophage supernatant. **E** The apoptosis assay of NSCLC cells (including A549, H1299 and H1650 cells) after treatment with macrophage supernatant. **F** The EdU staining of NSCLC cells (including A549, H1299 and H1650 cells) after treatment with macrophage supernatant. Error bars represent the means ± SEM of three independent experiments. PMA: phorbol 12-myristate 13-acetate; NSCLC: non-small cell lung cancer; Mac-S: macrophage supernatant. ^∗∗^*p* < 0.01. *N* = 8.

To initiate our investigation, we collected the culture medium of polarized macrophages (THP-1 cells after treatment with PMA) and co-cultured it with NSCLC cells (Fig. 1A). Figure 1B shows the typical exomes from the culture medium of polarized macrophages and the intensity. Subsequently, we conducted a range of assays, including cell proliferation, colony formation, apoptosis, and EdU staining to evaluate the impact of co-culturing on in vitro cell proliferation. Our study used three NSCLC cell lines: A549, H1299, and H1650. As illustrated in Fig. 1C–E, NSCLC cells co-cultured with macrophage supernatant showed enhanced proliferation, while apoptosis was inhibited in vitro. In addition, EdU staining demonstrated an increase in the proliferation of NSCLC cell lines after treatment with macrophage supernatant (Fig. 1F). Taken together, these results indicated that co-culturing with macrophages may promote the proliferation of NSCLC cells in vitro.

### LncRNA *NORAD* targeted *miR-520g-3p*/*SMIM22* in NSCLC cells

**Fig. 2:**
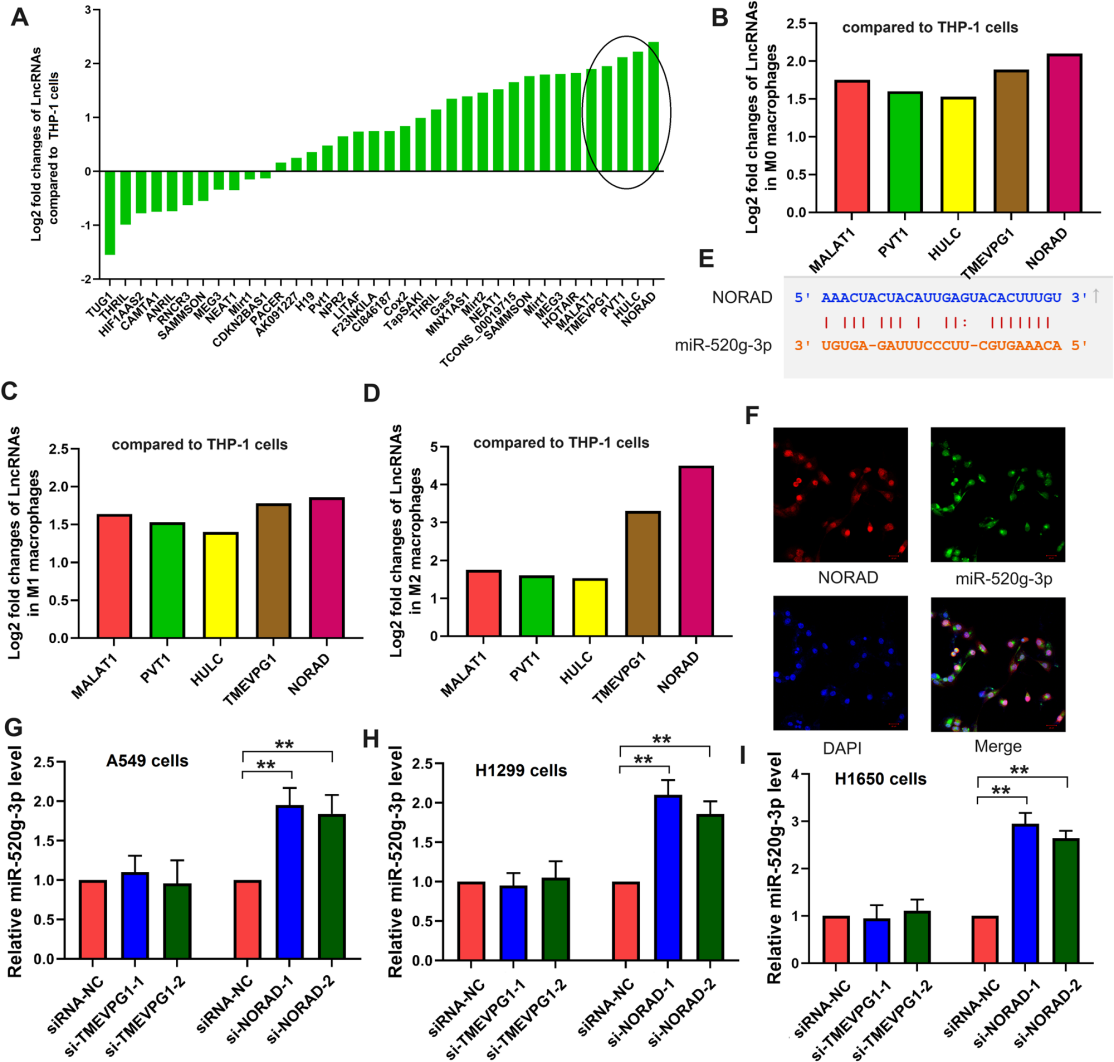
LncRNA *NORAD* targets *miR-520g-3p* in NSCLC cells. **(A)** The expression of certain lncRNAs in THP-1 cells treated with phorbol 12-myristate 13-acetate (PMA). **B**–**D** The expression of the top five lncRNAs in M0, M1, and M2 macrophages. **E** The potential binding site between lncRNA *NORAD* and *miR-520g-3p* was predicted using the TargetScan dataset. **F** FISH assay of lncRNA *NORAD* and *miR-520g-3p* in NSCLC cells. **G**–**I** The expression of *miR-520g-3p* in NSCLC cells after knockdown of lncRNA *NORAD*. Error bars represent the means ± SEM of three independent experiments. siRNA or si: Small interfering RNA; ^∗∗^*p* < 0.01.

LncRNAs play a significant role in various mechanisms of tumor progression^20–22^. We quantified the levels of various lncRNAs in polarized macrophages (Fig. 2A). The top five lncRNAs with the highest levels (i.e., *MALAT1*, *PVT1*, *HULC*, *TMEVPG1*, and *NORAD*) were selected for subsequent research. We then examined the levels of these lncRNAs in M0, M1, and M2 macrophages. As shown in Fig. 2B–D, only the levels of *TMEVPG1* and *NORAD* increased, with lncRNA *NORAD* showing the highest level in M2 macrophages. Overall, these data reveal that lncRNA *NORAD* is secreted by M2 macrophages in vitro.

Building upon these experiments, we explored the role of lncRNA *NORAD* in NSCLC. The potential binding site between lncRNA *OIP5-AS1* and *miR-520g-3p* was predicted using the TargetScan dataset (Fig. 2E). Subsequently, we treated A549 cells with a macrophage supernatant. FISH assay revealed that lncRNA *NORAD* co-localized with *miR-520g-3p* in A549 cells after co-culturing, suggesting that the function of lncRNA *NORAD* may be associated with *miR-520g-3p* (Fig. 2F). We also knocked down *NORAD* in three NSCLC cell lines, including A549, H1299, and H1650 cell lines. Using RT-PCR analysis, we found that the deficiency of lncRNA *NORAD* may up-regulate the RNA expression of *miR-520g-3p* in NSCLC cells (Fig. 2G–I). These results indicated that lncRNA *NORAD* may target *miR-520g-3p* in NSCLC cells.

**Fig. 3:**
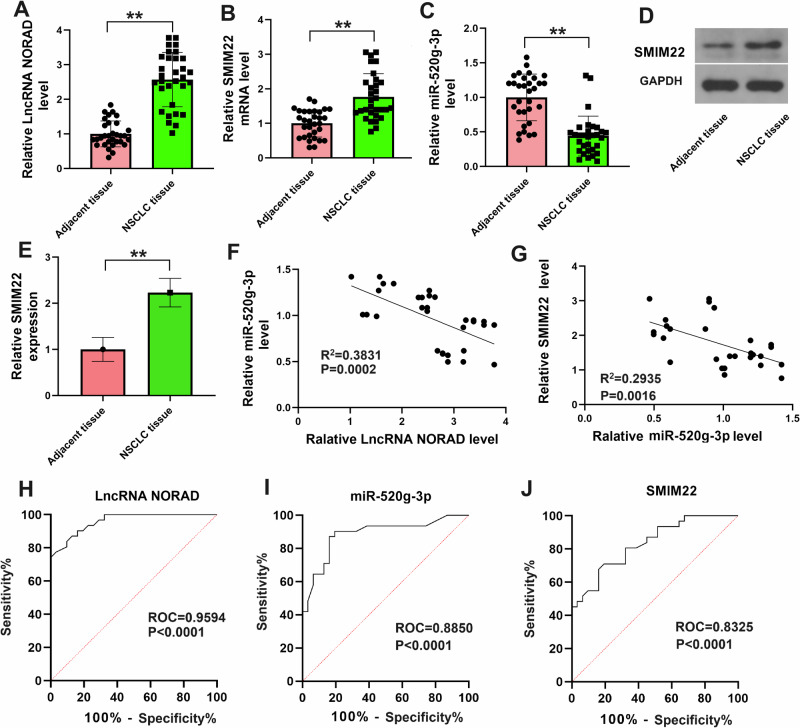
LncRNA *NORAD* targets *miR-520g-3p*/*SMIM22* in NSCLC tissues. **A**–**C** The levels of lncRNA *NORAD*, *SMIM22* mRNA, or *miR-520g-3p* level in adjacent tissue (*N* = 31) and NSCLC tissues (*N* = 31). **D**, **E** The protein level of SMIM22 in NSCLC tissues and adjacent tissues (*N* = 8). **F**, **G** The linear regression and correlation analyses between *miR-520g-3p* and lncRNA *NORAD*, or between *miR-520g-3p* and *SMIM22*. **H**–**J** The ROC curve of lncRNA *NORAD*, *miR-520g-3p*, or *SMIM22* in the NSCLC patients. Error bars represent the means ± SEM of three independent experiments. ROC: Receiver Operating Characteristic. ^∗∗^*p* < 0.01.

Histological evidence also supported the aforementioned results. As shown in Fig. 3A–E, in comparison to adjacent normal tissues, lncRNA *NORAD* and *SMIM22* were up-regulated, whereas *miR-520g-3p* was down-regulated in NSCLC tissues (N = 31). The protein level of SMIM22 in adjacent normal tissues and NSCLC tissues is shown in Fig. 3D, E. Linear regression and correlation analyses demonstrated a moderate correlation between *miR-520g-3p* levels in NSCLC tissues and the levels of lncRNA *NORAD* or *SMIM22* (Fig. 3F, G). We further evaluated the diagnostic potential of lncRNA *NORAD*, *miR-520g-3p*, or *SMIM22* in tissue samples as biomarkers for NSCLC using receiver operating characteristic (ROC) curve analysis from the NSCLC tissues. The results, as depicted in Fig. 3H–J, yielded area under the ROC curve (AUC) values of 0.9594, 0.8850, and 0.8325, respectively. In summary, these findings collectively illustrated that lncRNA *NORAD* targets *miR-520g-3p*/*SMIM22* in NSCLC cells.

### Identification of lncRNA NORAD in EVs in M2 macrophages

**Fig. 4:**
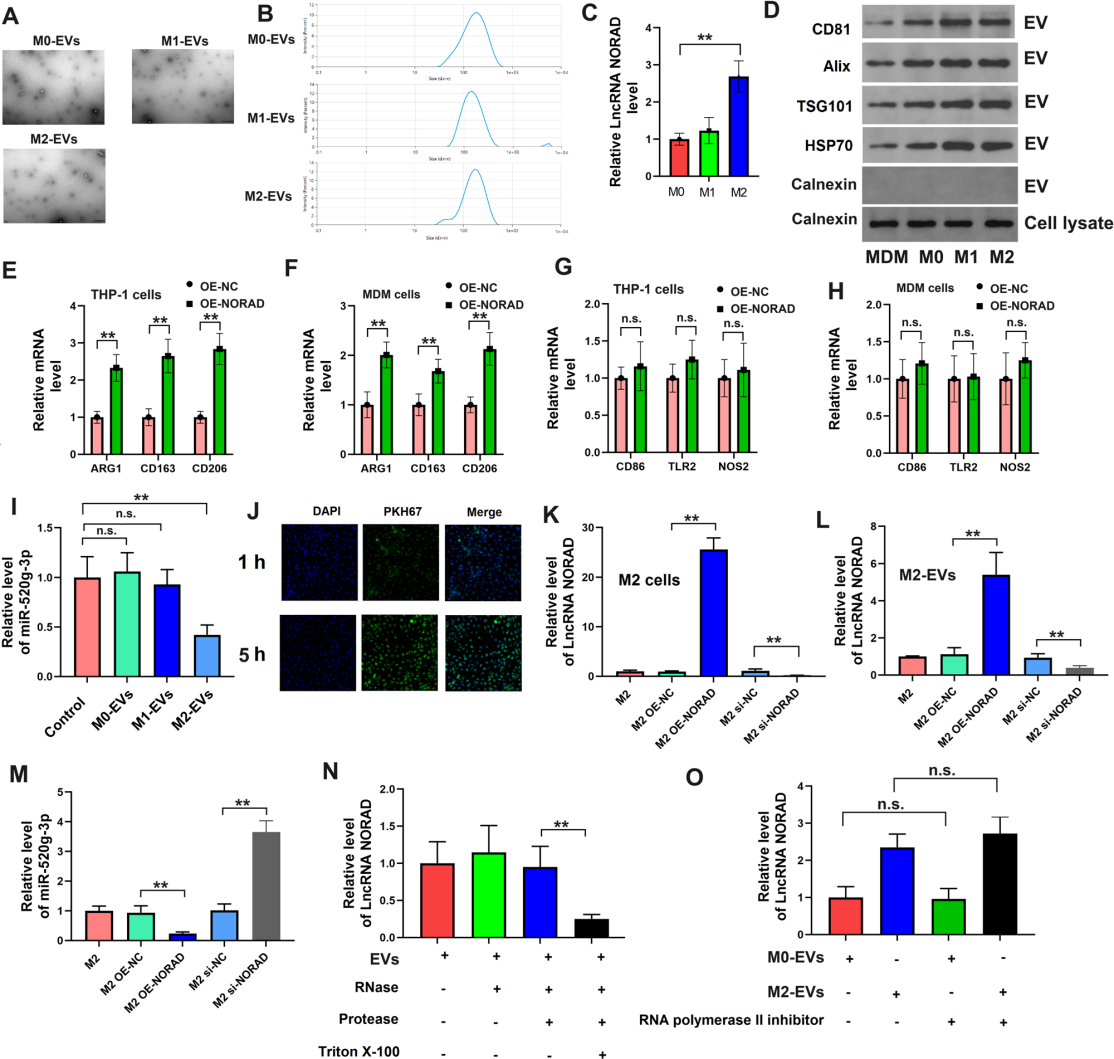
Identification of lncRNA NORAD in EVs in M2 macrophages. **A** EVs derived from THP-1 cells as identified by TEM. **B** EVs derived from THP-1 cells as identified by NTA. **C** The expression of lncRNA *NORAD* in M0, M1, and M2 macrophage-derived EVs. **D** The protein level of exosome markers (TSG101, Alix, HSP70, and CD81) in M0, M1, and M2 macrophage-derived EVs or MDM-derived EVs. **E**–**H** The mRNA level of M1 macrophage markers (CD86, TLR2, and NOS2) or M2 macrophage markers (ARG1, CD163, and CD206) in THP-1 cell-derived EVs or MDM cells. **I** The expression of *miR-520g-3p* in NSCLC cells after treatment with different macrophage-derived EVs. **J** Immunofluorescence of PKH67 in NSCLC cells. **K**, **L** The expression of lncRNA *NORAD* in M2 macrophages or M2 macrophage-derived EVs. **M** The expression of *miR-520g-3p* in NSCLC cells after treatment with different groups of M2 macrophage-derived EVs. **N**, **O** The expression of lncRNA *NORAD* in NSCLC cells after treatment with M2 macrophage-derived EVs and inhibitors. Error bars represent the means ± SEM of three independent experiments. EVs: extracellular vesicles; MDM: monocyte-derived macrophage; OE: over-expression; NC: negative control. ^∗∗^*p* < 0.01.n,s., not significant.

Given that lncRNA *NORAD* is secreted by M2 macrophages in supernatant, we subsequently investigated whether lncRNA *NORAD* was secreted from M2 macrophages via EVs. Here, we polarized THP-1 cells to M0 macrophages, M1 macrophages, and M2 macrophages, over-expressed lncRNA *NORAD* in THP-1 cells or monocyte-derived macrophage (MDM) cells, and collected the EVs from them. The EVs were identified by TEM and NTA (Fig. 4A, B). We found that lncRNA *NORAD* expression in M2 macrophage EVs was the highest (Fig. 4C). Furthermore, the western blotting results revealed a substantial up-regulation of EVs markers (TSG101, Alix, HSP70, and CD81) in different groups of EVs (Fig. 4D). So, we over-expressing lncRNA *NORAD* in M2 macrophages. As shown in Fig. 4E–H, after over-expressing lncRNA *NORAD*, the EVs from THP-1 cells or MDM cells exhibited an obvious increase in M2 macrophage marker expression (ARG1, CD163, and CD206). However, M1 macrophage markers (CD86, TLR2, and NOS2) did not show significant alterations. Moreover, we also quantified the expression of lncRNA *NORAD* in EVs from M0 macrophages, M1 macrophages, and M2 macrophages. These results indicated the potential M2 macrophage characteristics in EVs.

Subsequently, we co-cultured A549 cells with EVs from M0, M1 or M2 macrophages for 5 h, then measured the levels of *miR-520g-3p*. The results showed that compared to Control, the levels of *miR-520g-3p* in the A549 cells treated with EVs from M0 or M1 macrophages were not significantly changed (*P* > 0.05, Fig. 4I), only significantly decreased in the A549 cells treated with EVs from M2 macrophages (*P* < 0.01, Fig. 4I). The PKH67 staining showed that many EVs were absorbed by A549 cells (Fig. 4J). We knocked down or over-expressed lncRNA *NORAD* in M2 macrophages (Fig. 4K, L). After treatment with EVs from lncRNA *NORAD* knockdown M2 macrophages, the expression of *miR-520g-3p* was up-regulated in NSCLC cells (Fig. 4M). However, the expression of *miR-520g-3p* was down-regulated in NSCLC cells after treatment with EVs from lncRNA *NORAD* over-expressed M2 macrophages. Furthermore, we found that RNase or protease did not affect the expression of lncRNA *NORAD* in NSCLC cells after co-culturing with M2 macrophage EVs (Fig. 4N). The RNA polymerase II inhibitor also could not inhibit the expression of lncRNA *NORAD* in NSCLC cells after co-culturing with M0 macrophage or M2 macrophage EVs (Fig. 4O). These findings indicated that the high expression of lncRNA *NORAD* in NSCLC cells was caused by M2 macrophage EVs, rather than being secreted by the cells themselves.

### *SMIM22*/*GALE* promotes glycolysis and proliferation in NSCLC cells

**Fig. 5:**
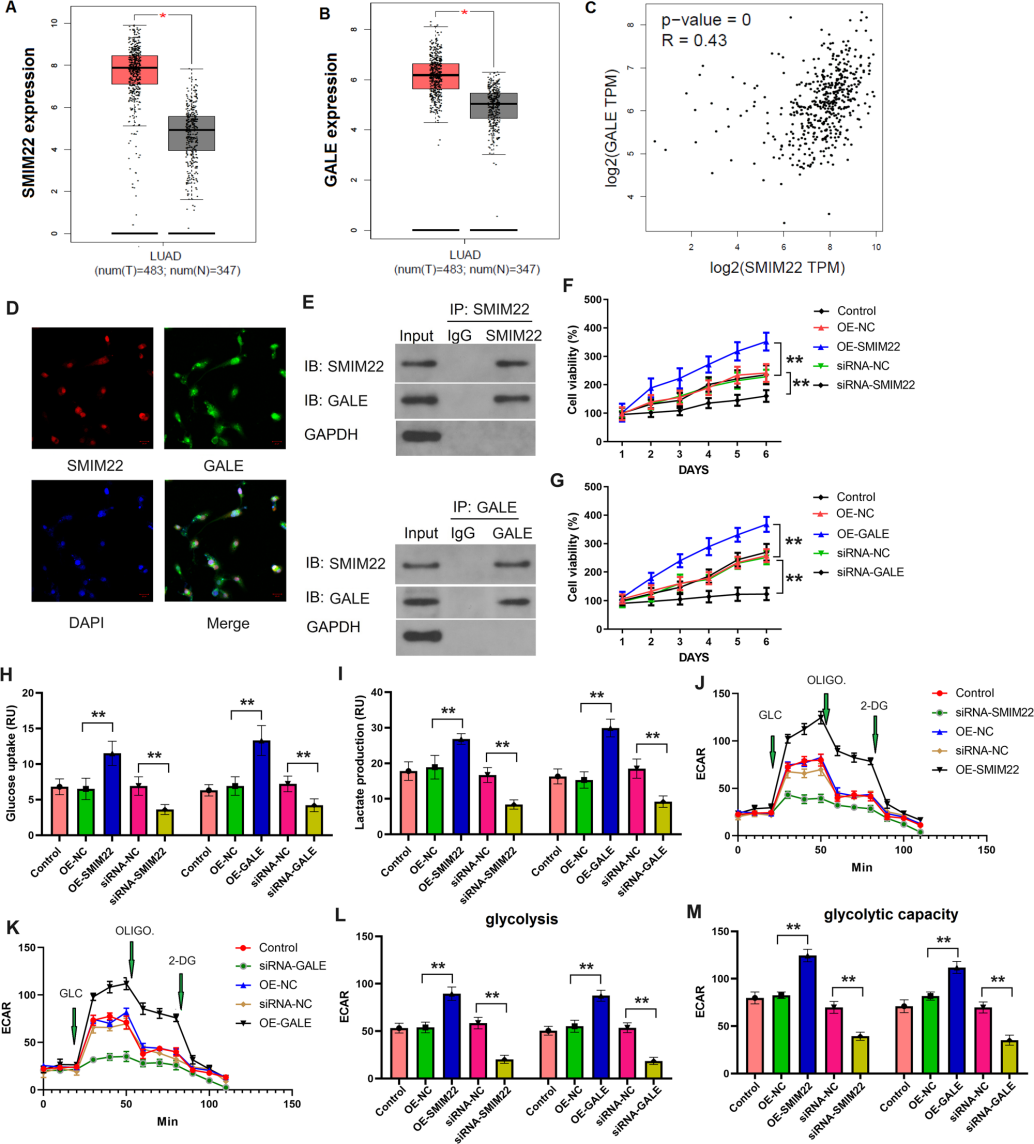
*SMIM22*/*GALE* promotes glycolysis and proliferation of NSCLC cells. **A**, **B** GEPIA dataset of *SMIM22* or *GALE* expression in NSCLC cases (*N* = 483) and controls (*N* = 347). **C** The linear regression and correlation analyses between *SMIM22* and *GALE* in NSCLC cases. **D** Immunofluorescence of *SMIM22* and *GALE* in NSCLC cells. **E** Immunoprecipitation assay suggests an interaction between *SMIM22* and *GALE*. **F**, **G** The cell viability of A549 cells after knockdown or over-expressed *SMIM22* or *GALE*. **H** Glucose uptake of A549 cells after knockdown or over-expressed *SMIM22* or *GALE*. **I** Lactate production of A549 cells after knockdown or over-expressed *SMIM22* or *GALE*. **J**–**M** The ECAR of A549 cells after knockdown or over-expressed *SMIM22* or *GALE*. Error bars represent the means ± SEM of three independent experiments. ^∗∗^*p* < 0.01.

To commence our investigation, we accessed RNA sequencing data from the GEPIA dataset, comprising 483 NSCLC cases and 347 control samples. Intriguingly, both *SMIM22* and *GALE* exhibited significant over-expression in NSCLC samples (Fig. 5A, B). Subsequently, we explored the relationship between *SMIM22* and *GALE* in NSCLC. Our bioinformatic analysis indicated a potential association between *SMIM22* and *GALE* in NSCLC tissues (Fig. 5C). Furthermore, immunofluorescence demonstrated the co-localization of *SMIM22* with *GALE* within the nucleus of NSCLC samples (Fig. 5D). To explore whether *SMIM22* physically interacted with *GALE*, potentially regulating target genes within the nucleus, we conducted an IP assay using specific antibodies against *SMIM22* or *GALE* in NSCLC tissues. The results revealed that *SMIM22* could be co-immunoprecipitated with *GALE* in tissues, suggesting a tangible interaction between *SMIM22* and *GALE* (Fig. 5E). In summary, these findings collectively underscore the over-expression of *SMIM22* and *GALE*, with *SMIM22* interacting with *GALE* in NSCLC tissues.

We delved further into the role of *SMIM22*/*GALE* in the progression of NSCLC, using the A549 cell line as research subjects. As shown in Fig. 5F, G, over-expressed *SMIM22* or *GALE* promoted the proliferation of NSCLC cells. Conversely, the knockdown of *SMIM22* or *GALE* inhibited the proliferation of NSCLC cells. Notably, cancer cells exhibit a preference for glycolysis as their primary mode of glucose catabolism, even in the presence of sufficient oxygen, a phenomenon commonly referred to as the Warburg effect^45^. Our observations revealed that the depletion of *SMIM22* or *GALE* in NSCLC cells led to a reduction in both lactate production and glucose uptake, signifying the suppression of glycolysis in these cells (Fig. 5H, I). Conversely, the introduction of *SMIM22* or *GALE* via ectopic expression resulted in elevated lactate production and glucose uptake. Furthermore, ECAR measurements confirmed that *SMIM22* or *GALE* knockdown led to a decrease in ECAR, while their over-expression increased ECAR (Fig. 5J–M). In summary, our findings provide compelling evidence for the role of *SMIM22*/*GALE* in promoting glycolysis and the proliferation of NSCLC cells.

### LncRNA NORAD in EVs promotes glycolysis through the *miR-520g-3p* axis in NSCLC cells

**Fig. 6:**
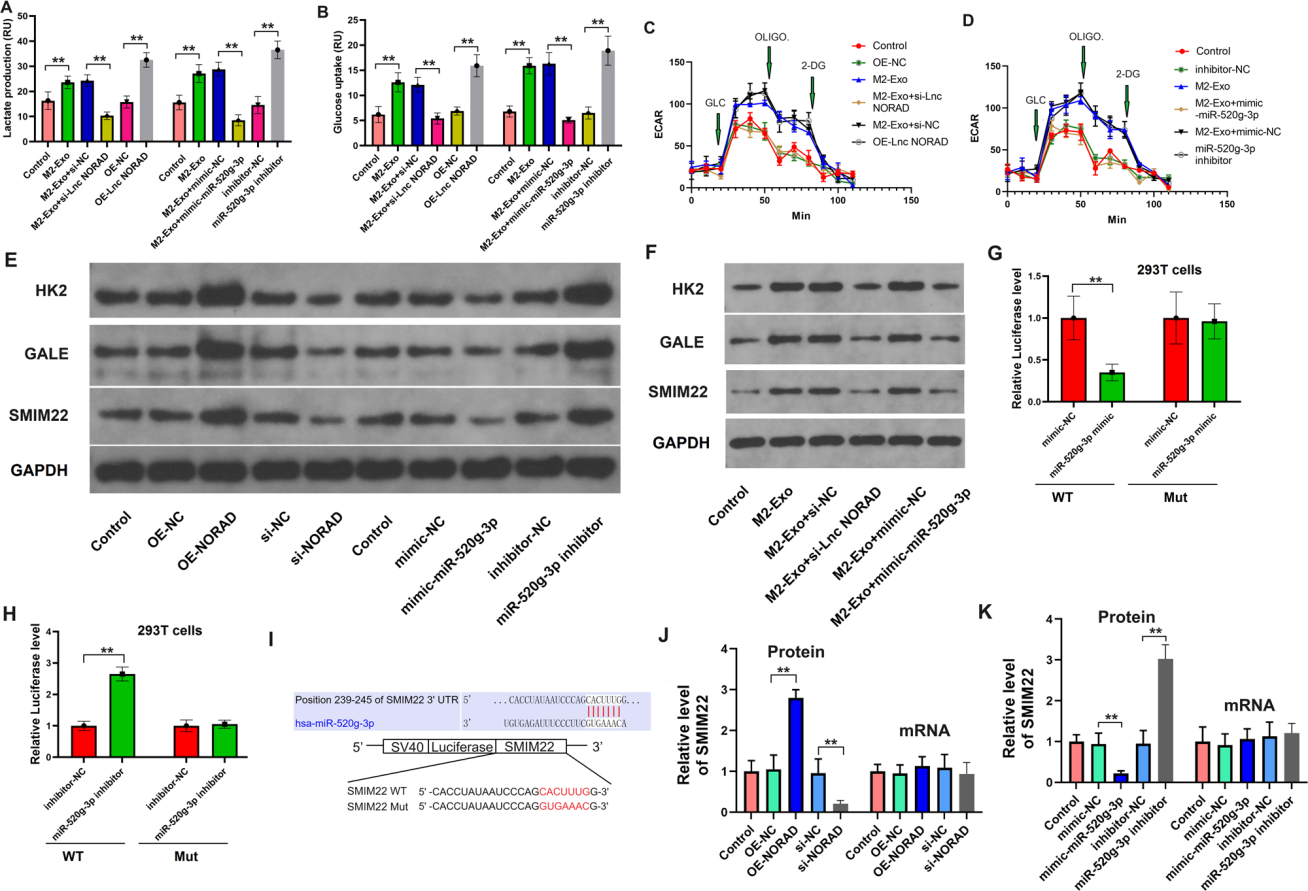
LncRNA NORAD in EVs promotes glycolysis through the *miR-520g-3p* axis in NSCLC cells. **A** Lactate production of A549 cells after treatment with different groups of EVs. **B** Glucose uptake of A549 cells after treatment with different groups of EVs. **C**, **D** The ECAR of A549 cells after treatment with different groups of EVs. **E**, **F** The expression of glycolysis-related marker (HK2), SMIM22 and GALE in different groups of NSCLC cells. **G**, **H** Dual-luciferase reporter assay confirms the direct binding between *miR-200c-3p* and the *SMIM22* 3’UTR regions. **I** The potential binding site between *SMIM22* and *miR-520g-3p* as predicted using the TargetScan dataset. **J**, **K** The expression of *SMIM22* in different groups of NSCLC cells. Error bars represent the means ± SEM of three independent experiments. ^∗∗^*p* < 0.01. *N* = 8.

We also examined the glycolysis of NSCLC cells after treatment with different groups of EVs. As shown in Fig. 6A, B, M2 macrophage-derived EVs or a *miR-520g-3p* inhibitor led to an increase in both lactate production and glucose uptake, particularly after the over-expression of lncRNA *NORAD* in EVs. Conversely, the knockdown of lncRNA *NORAD* or transfection of *miR-200c-3p* mimics in EVs restored the impact of lactate production and glucose uptake of cell-derived EVs after co-culturing. ECAR measurements further supported these conclusions (Fig. 6C, D). Western blotting analysis also demonstrated that the knockdown of lncRNA *NORAD* or the transfection of *miR-200c-3p* mimics decreased the protein levels of glycolysis-related proteins (HK2, GALE, and SMIM22) in NSCLC cells, while the *miR-200c-3p* inhibitor could increase the protein levels of these markers (Fig. 6E, F). These results suggested that lncRNA *NORAD* may promote glycolysis in vitro, which is negatively regulated by *miR-520g-3p*. These findings indicate that lncRNA NORAD in EVs derived from M2 macrophages promotes glycolysis through the *miR-520g-3p* axis in NSCLC cells.

### LncRNA NORAD in EVs targets *SMIM22* and *miR-520g-3p* in NSCLC cells

To confirm the direct interaction between *miR-520g-3p* and the *SMIM22* 3′UTR regions, we performed a luciferase reporter assay using plasmids containing the WT and MUT *SMIM22* 3’UTR sites with the *miR-520g-3p* binding site. Upon transfection with *miR-520g-3p* mimics and treatment with a *miR-520g-3p* inhibitor, we observed a noticeable reduction in luciferase activity in comparison to cells transfected with control mimics and those treated with a control inhibitor, respectively (Fig. 6G, H). The potential binding site between *SMIM22* and *miR-520g-3p* was predicted using the TargetScan dataset (Fig. 6I). As lncRNA was found to interact with *miR-520g-3p* and *SMIM22* in NSCLC tissues, we evaluated the protein level of SMIM22 in each group of NSCLC cell lines. The results demonstrated that the over-expression of lncRNA *NORAD* or treatment with a *miR-520g-3p* inhibitor increased the level of SMIM22, while the knockdown of lncRNA *NORAD* or *miR-520g-3p* mimics inhibited the level of SMIM22 in vitro (Fig. 6J, K). Together, these pieces of evidence underscore that lncRNA NORAD in EVs derived from M2 macrophages targets SMIM22 and *miR-520g-3p*.

### LncRNA NORAD in EVs promotes NSCLC cell proliferation through the *miR-520g-3p* axis

**Fig. 7:**
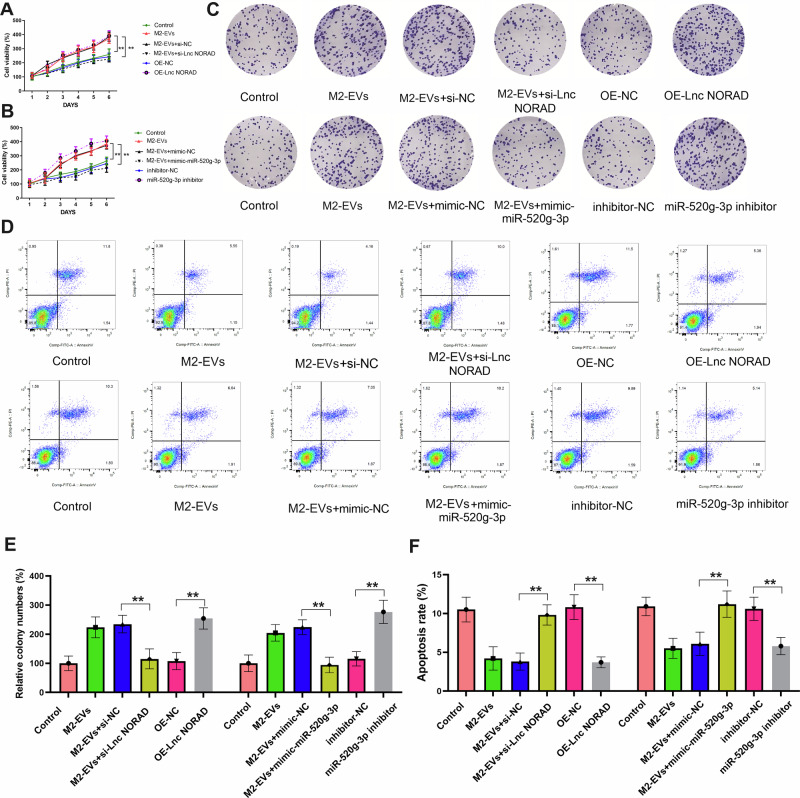
LncRNA NORAD in EVs promotes NSCLC cell proliferation through the *miR-520g-3p* axis. The cell viability (**A**, **B**), colony formation (**C**, **E**), and apoptosis assay (**D**, **F**) of A549 cells after treatment with different groups of EVs. Error bars represent the means ± SEM of three independent experiments. ^∗∗^*p* < 0.01. *N* = 8.

To elucidate the role of lncRNA NORAD in EVs in NSCLC, different groups of EVs derived from M2 macrophages were isolated and co-cultured with NSCLC cell lines. Subsequently, functional experiments were performed to examine the effect of M2 macrophage-derived lncRNA NORAD in EVs on NSCLC cell proliferation in vitro. As depicted in Fig. 7A, B, D, F, NSCLC cells treated with M2 macrophage-derived EVs exhibited enhanced proliferation and inhibited apoptosis. However, upon the removal of EVs from the cultured medium, the promotion of proliferation and inhibition of apoptosis effects were reversed. These results suggested that M2 macrophage-derived EVs promote cell proliferation and inhibit apoptosis in vitro. Nevertheless, after knocking down lncRNA *NORAD* or transfecting *miR-520g-3p* mimics into EVs, these effects disappeared. Additionally, colony formation assays showed that NSCLC cell proliferation significantly increased after treatment with M2 macrophage-derived EVs (Fig. 7C, E). However, the proliferation of NSCLC cells was restored after treatment with si-lncRNA *NORAD* in EVs, and *miR-520g-3p* mimics could promote the proliferation of NSCLC cells again. Taken together, these results indicate that lncRNA NORAD in EVs promoted NSCLC cell proliferation through the *miR-520g-3p* axis.

### LncRNA NORAD in EVs promotes NSCLC tumor growth through the *miR-520g-3p* axis in vivo

**Fig. 8:**
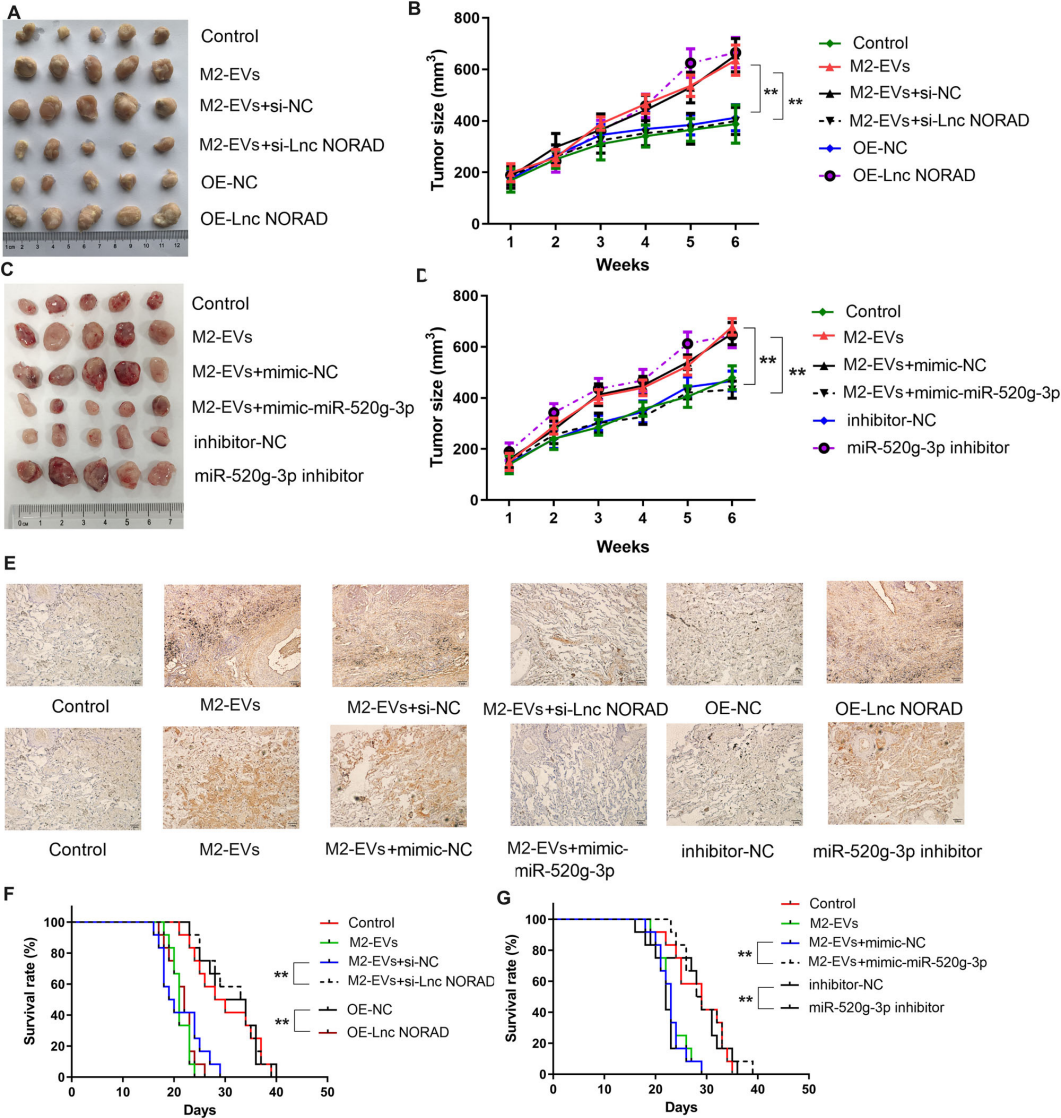
LncRNA NORAD in EVs promotes NSCLC tumor growth through the *miR-520g-3p* axis in vivo. **A**–**D** The tumor size of xenograft tumor samples in animal models after treatment with different groups of EVs. **E** Immunohistochemistry staining of Ki-67 in xenograft tumor tissues. **F**, **G** The percentage survival of xenograft tumor mice after treatment with different groups of EVs. Error bars represent the means ± SEM of three independent experiments. ^∗∗^*p* < 0.01. *N* = 5.

We established mouse xenograft tumor models (*N* = 5) using the LL2 cell line. Various groups of EVs were intravenously administered to the mice every three days. After six rounds of injections, the mice were euthanized on day 30, and the tumors were collected. As anticipated, EVs derived from M2 macrophages substantially increased both the volume and weight of the xenograft tumors. In contrast, the control group and the group receiving EVs with si-lncRNA *NORAD* exhibited a reversal of the tumor growth acceleration induced by M2 macrophage-derived EVs (Fig. 8A, B). The *miR-520g-3p* inhibitor could also alleviate the xenograft tumor growth induced by M2 macrophage-derived EVs in vivo (Fig. 8C, D). Immunohistochemistry staining of Ki-67 in NSCLC showed a similar result (Fig. 8E). Moreover, M2 macrophage-derived exosome injection decreased the percentage survival of xenograft tumor mice, and EVs with si-lncRNA *NORAD* or *miR-520g-3p* inhibitor could restore the percentage survival in vivo (Fig. 8F, G). These findings are consistent with the in vitro results showing that M2 macrophages-derived lncRNA NORAD in EVs promoted NSCLC tumor growth through the *miR-520g-3p* axis in vivo.

## Discussion

LC, a prevalent malignant disease, is characterized by a dismal prognosis and high morbidity. NSCLC, the most common form of LC, constitutes ~85% of cases^46^. Smoking is well-recognized as the primary causative factor for NSCLC^47^. A significant challenge in NSCLC management is the absence of discernible clinical symptoms and ineffective screening programs, leading to the majority of patients being diagnosed at advanced stages with a bleak prognosis^48^. Our findings demonstrate that co-culturing with macrophages promotes the proliferation of NSCLC cells. Furthermore, our investigations reveal that lncRNA NORAD in EVs, derived from M2 macrophages, may play a critical role in the progression of NSCLC. These insights suggest that lncRNA *NORAD* holds promise as a potential biomarker for NSCLC diagnosis and therapeutic intervention.

Metabolic reprogramming stands as a hallmark of cancer, with glycolysis playing a pivotal role in various malignancies. The Warburg effect, often referred to as aerobic glycolysis, is a phenomenon where cancer cells demonstrate a predilection for utilizing glucose as their primary energy source over oxygen. This metabolic preference fulfils the energy demands required for unregulated cell proliferation^49,50^. Emerging evidence has established aerobic glycolysis as a metabolic hallmark of malignant tumor cells, fostering tumorigenicity^51^. Recent articles have reported several lncRNAs, such as *lncRNA-AC020978*^52^, *LINC01123*^53^, and *ABHD11-AS1*^54^, in promoting glycolysis rates, thereby supporting cell growth and driving tumor progression in NSCLC. Targeting cancer cell glycolytic metabolism presents a promising avenue for therapeutic interventions. Our study underscores the role of lncRNA *NORAD* in promoting tumor malignancy and modulating energy homeostasis. This is accomplished by elevating the expression of glycolytic genes and the activity of related enzymes, resulting in an overall increase in glycolysis in a laboratory setting. Specifically, lncRNA NORAD in EVs is shown to promote glycolysis through the *miR-520g-3p* axis in NSCLC cells. Furthermore, our investigations reveal that *SMIM22*/*GALE* may enhance glycolysis and cell proliferation in NSCLC. Given that *SMIM22* is targeted by lncRNA NORAD in EVs in vitro, it is plausible to speculate that lncRNA NORAD in EVs influences the expression of *SMIM22*/*GALE* through the *miR-520g-3p* axis, thereby accelerating glycolysis in NSCLC cells.

Exosomes, small extracellular nano-vesicles, play a crucial role in intercellular communication by transporting vesicle contents, including RNAs and proteins^55^. EVs derived from various cell types can be detected in bodily fluids such as blood, urine, saliva, and ascites^56^. A growing body of evidence supports the essential involvement of EVs in various biological processes, particularly tumorigenesis^57^. For instance, EV *circ_0014235* has been implicated in enhancing cisplatin resistance and promoting NSCLC development by modulating the *miR-520a-5p*/CDK4 signaling pathway^58^. These findings underscore the pervasive role of EVs as regulatory factors in tumor development. In our study, we establish that lncRNA *NORAD* is abundant in EVs derived from M2 macrophages, which, in turn, enhances glycolysis and proliferation of NSCLC cells through the *miR-520g-3p* axis in vitro. Furthermore, lncRNA NORAD in EVs derived from M2 macrophages is found to promote NSCLC tumor growth through the *miR-520g-3p* axis in vivo. These discoveries shed light on the involvement of M2 macrophage-derived EVs in the mechanisms underlying NSCLC.

LncRNAs, characterized as RNAs exceeding 200 nucleotides in length, participate in a diverse range of cellular processes, including cell proliferation, autophagy, apoptosis, and senescence^59,60^. These lncRNAs exert their regulatory functions by interacting with miRNAs^61,62^. Among these lncRNAs, lncRNA *NORAD* stands out as a recently identified RNA molecule activated in response to DNA damage. It plays a crucial role in maintaining genomic stability and orchestrating regular mitosis^23,63^. LncRNA *NORAD* is highly expressed and conserved across mammalian species, with the human genome hosting the *NORAD* gene. This gene gives rise to a 5.3 kb transcript located at *Chr20q11.23*^64^. The transcripts of lncRNA *NORAD* are distributed in both the cytoplasm and the nucleus^63,65^. Notably, lncRNA *NORAD* has been found to specifically interact with numerous proteins, including the IL-8 transcriptional repressor SFPQ (i.e., the splicing factor proline and glutamine-rich protein). Of these proteins, 71% are localized in the nucleus, with only 5% found in the cytoplasm. Many of these proteins play vital roles in DNA replication and repair within the nucleus^65^. Inside the nucleus, lncRNA *NORAD* has been shown to interact with *RBMX* (i.e., the RNA-binding motif protein, X-linked gene) and participate in the assembly of a topoisomerase complex known as the *NORAD*-activated ribonucleoprotein complex 1. This complex is closely linked to genome stability^65^. In the cytoplasm, lncRNA *NORAD* binds to PUMILIO (i.e., an RNA-binding protein) and influences the mRNA levels of PUMILIO targets, many of which are associated with genes governing cell proliferation and division^63^. Deletion of lncRNA *NORAD* in knockout mice results in the overactivity of PUMILIO, inhibiting genes essential for normal mitosis and leading to the accumulation of aneuploid cells. The absence of lncRNA *NORAD* also affects genes responsible for maintaining mitochondrial homeostasis, causing significant mitochondrial dysfunction. Intriguingly, knockout mice lacking lncRNA *NORAD* exhibit a multisystem degenerative phenotype reminiscent of premature aging. To date, research on the role of lncRNA *NORAD* in various diseases has primarily focused on its impact on different types of cancer, including ovarian cancer, gastric cancer, pancreatic cancer, and colorectal cancer^24,25,66,67^. However, there have been no prior reports regarding the influence of lncRNA *NORAD* on NSCLC. In our research, we demonstrate that lncRNA *NORAD* is enriched in EVs derived from THP-1 polarized M2 macrophages. Our findings also indicate that lncRNA NORAD in EVs may modulate *SMIM22* and *miR-520g-3p* in NSCLC cells. Additionally, lncRNA NORAD in EVs is found to enhance glycolysis and proliferation of NSCLC cells through the *miR-520g-3p* axis in vitro. Notably, our study reveals that lncRNA NORAD in EVs promotes NSCLC tumor growth through the *miR-520g-3p* axis in vivo.

There were several limitations in our studies. First, A549 cells are used as the research subject of NSCLC cancer line. We will use actually NSCLC primary cells in our future researches. Moreover, these potential biomarkers, such as *miR-520g-3p* and lncRNA *NORAD* were difficult to be used to diagnose or in therapy of NSCLC, owing to the astriction of miRNA and lncRNA detection. The roles of targeted molecules have received increasing attention, but its effects and underlying mechanism are currently unclear. The involvement of EVs in the mechanisms of NSCLC is still worth exploring, and the results would assist us to better recognize the roles of EVs in NSCLC progression. It will be interesting to see how to optimally utilized non-coding RNA targeted molecules to achieve the better therapeutic benefits in NSCLC.

In conclusion, our research underscores the potential role of M2 macrophage-derived lncRNA NORAD in EVs in NSCLC. We found that *SMIM22*/*GALE* promoted glycolysis and proliferation of NSCLC cells. Moreover, lncRNA NORAD in EVs targeted *SMIM22* and *miR-520g-3p* in NSCLC cells. Notably, lncRNA NORAD in EVs promoted NSCLC cell proliferation, and promoted NSCLC tumor growth through the *miR-520g-3p* axis. These findings highlighted that M2 macrophage-derived lncRNA NORAD in EVs may participate in the progression of NSCLC.

### Supplementary information

Supplementary information file

## Data Availability

The raw data supporting the conclusions of this article will be made available from the authors, without undue reservation.
