# Supplementary material for: M2 macrophage-derived lncRNA NORAD in EVs promotes NSCLC progression via miR-520g-3p/SMIM22/GALE axis
Source: NPJ Precis Oncol. 2024 Aug 30;8:185. doi: 10.1038/s41698-024-00675-x (PMC11364787; doi:10.1038/s41698-024-00675-x)
Supplement: Supplementary file 1 — Supplementary information file [file 41698_2024_675_MOESM1_ESM.pdf]

Supplementary Table 1. Clinicopathological factors of samples of patients with non-small lung cell cancer

Characteristic	N (Total=31)	Percentage
**Age(years)**		
≥ 60	19	61.29%
< 60	11	35.48%
**Gender**		
Male	17	54.84%
Female	14	45.16%
**Smoking status**		
Never	4	12.90%
Former	7	22.58%
Current	20	64.52%
**T stage**		
T1	15	48.39%
T2	10	32.26%
T3	4	12.90%
T4	2	6.45%
**N stage**		
N0	18	58.06%
N1	10	32.26%
N2	3	9.68%
N3	0	0.00%
**M stage**		
M0	31	100.00%
M1	0	0.00%
**Histological types**		
SCC	13	41.94%
ADC	18	58.06%

SCC: squamous cell carcinoma; ADC: adenocarcinoma.

Supplementary Table 2. Antibodies used in the western blot analysis.

Reagent	Source	Identifier
Primary antibodies		
Anti-SMIM22 antibody, rabbit monoclonal	Abcam	ab252833
Anti-CD81 antibody, rabbit monoclonal	Abcam	ab197896
Anti-Alix antibody, rabbit polyclonal	Abcam	ab237704
Anti-TSG101 antibody, rabbit monoclonal	Abcam	ab308304
Anti-HSP70x antibody, rabbit monoclonal	Cell Signaling Technology	#3771

Anti-HK2 antibody, rabbit monoclonal	Cell Signaling Technology	#3230
Anti-GALE antibody, rabbit polyclonal	Cell Signaling Technology	#9145
Anti-GAPDH antibody, rabbit polyclonal	Abcam	ab9485
Anti-Calnexin antibody, rabbit polyclonal	Abcam	ab22595
Secondary antibodies		
Goat Anti-Rabbit IgG H&L (HRP)	Abcam	ab97051

Supplementary Table 3. Primers used in the RT-PCR.

**RT-PCR Primers**		
SMIM22	GGAAAGAACACCCGGAAATGA	GTGCTGCAACTGGGTAAACAC
LncRNA NORAD	TCACCAAGTTTGGACACCGT	ATAGTGGGGCAGGTCCTTCT
miR-520g-3p	GTAGTGGTGGCCTCTTGGAC	GAGGAGTGGGTGTTGGGAGA
ARG1	ACTTAAAGAACAAGAGTGTGATGTG	GTCCACGTCTCTCAAGCCAA
CD163	CTTGGGACTTGGACGATGCT	GGTATCTTAAAGGCTCACTGGGT
CD206	GCCTCGTTGTTTTGCGTCTT	GAGAACAGCACCCGGAATGA
CD86	TTCCTGCTCTCTGCTAACTTCAG	TCCCTCTCCATTGTGTTGGT
TLR2	GGCGTTCTCTCAGGTGACTG	CCCTGTCTTCCTGCCTTCAC
NOS2	TCCAAGGTATCCTGGAGCGA	CAGGGACGGGAACTCCTCTA
GALE	GGTTCATTCATTGCCGGCCT	ACTTGGACTTGCCGTAAGGG
GAPDH	AGTGTGACGTGGACATCCGCA	ATCCACATCTGCTGGAAGGTGGAC

Supplementary Figure 1. The gating strategy in flow cytometry analysis.

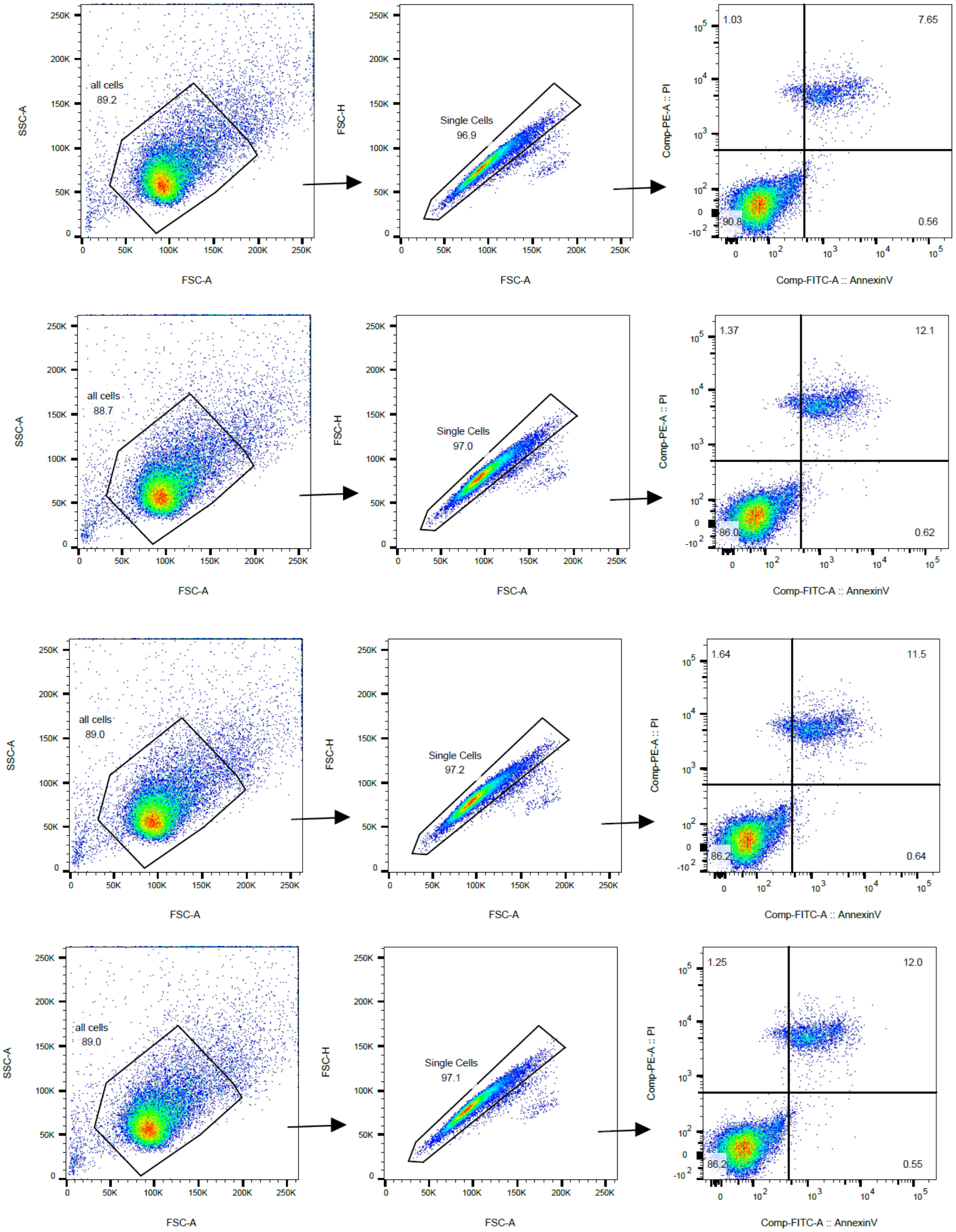

Supplementary Figure 2. Uncropped and unprocessed scans of blots used in the manuscript.

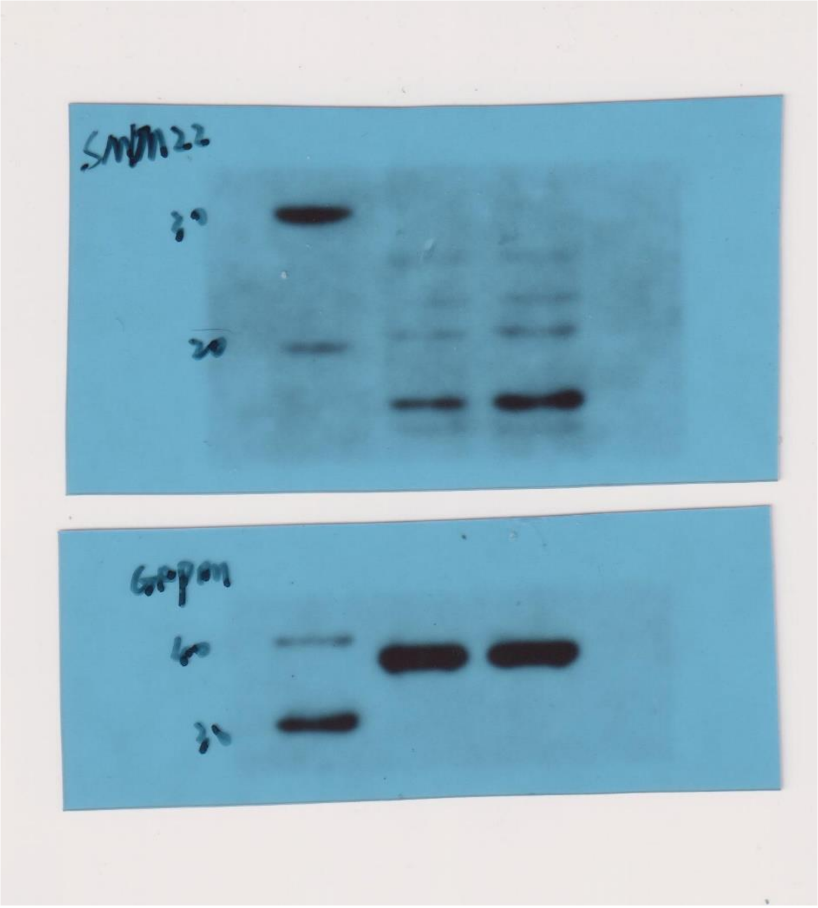

Fig. 3D

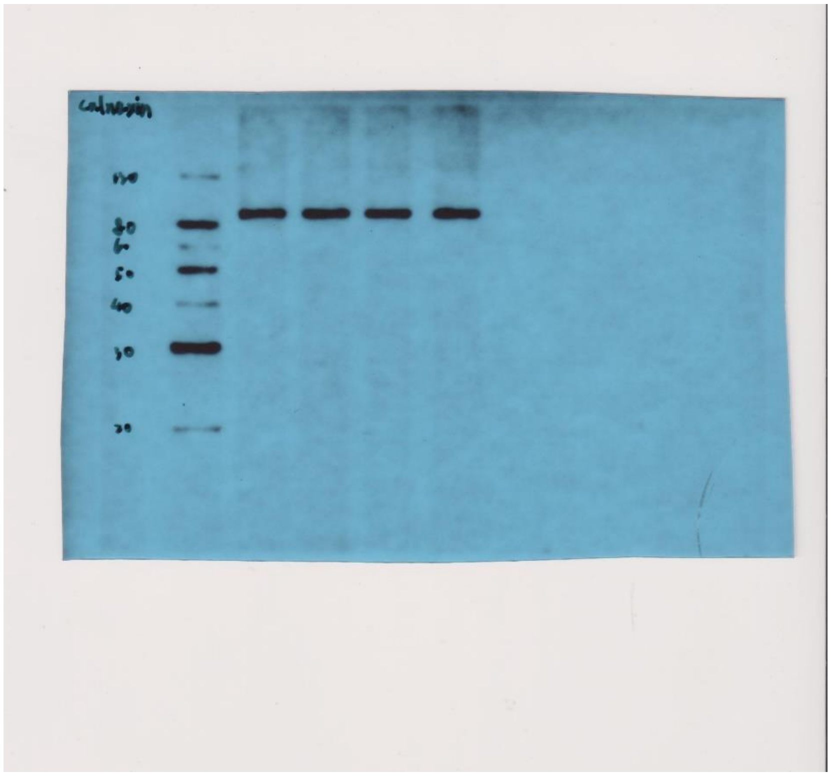

Fig. 4H Calnexin (negative control)

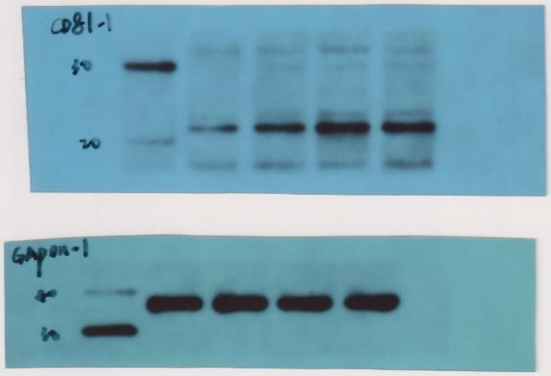
CD81-1
GAPDH-1

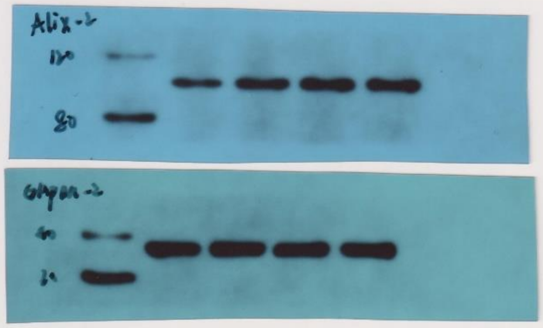
Alix-2
120
80
GAPDH-2

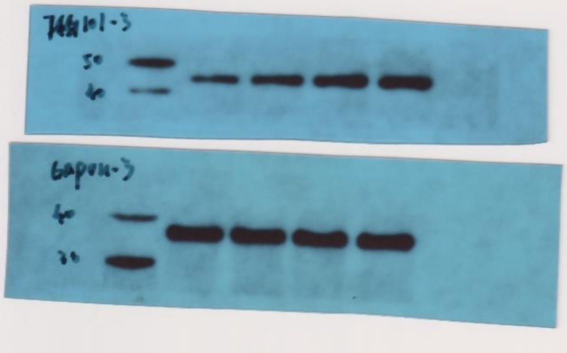
TSG101-3
50
40
GAPDH-3
40
30

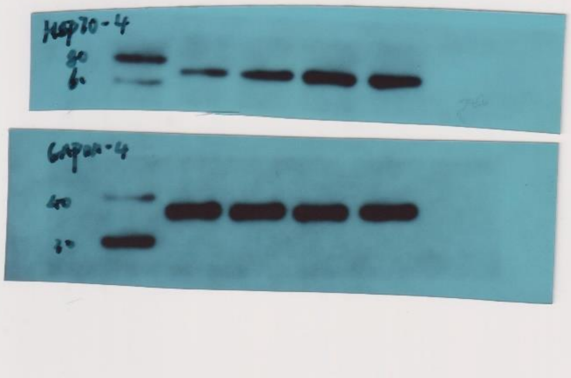
HSP70-4
80
60
GAPDH-4

Fig. 4H

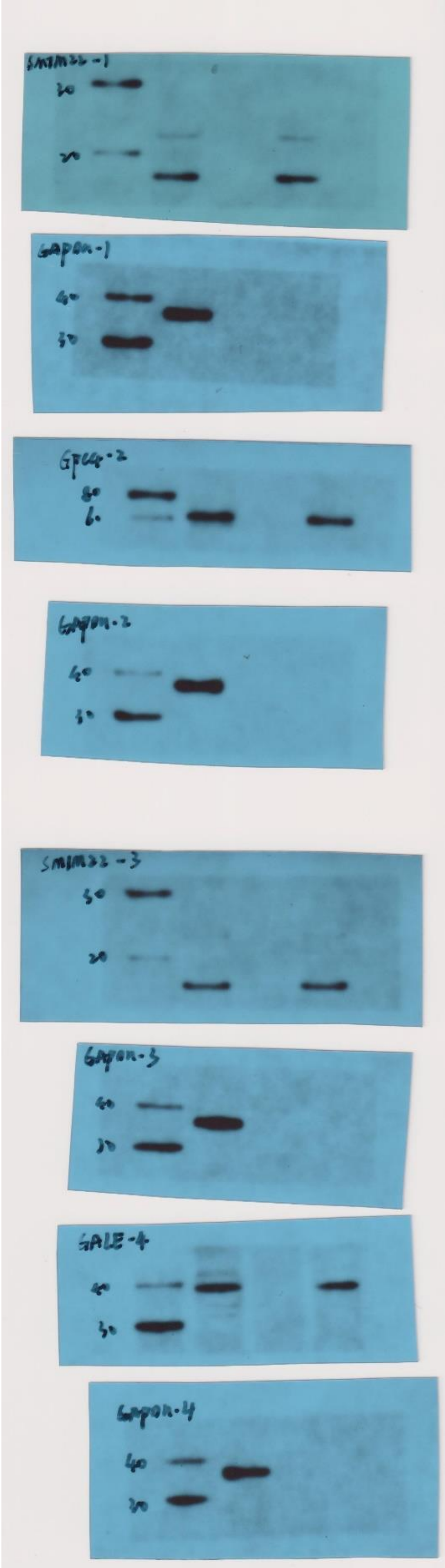

Fig.5E

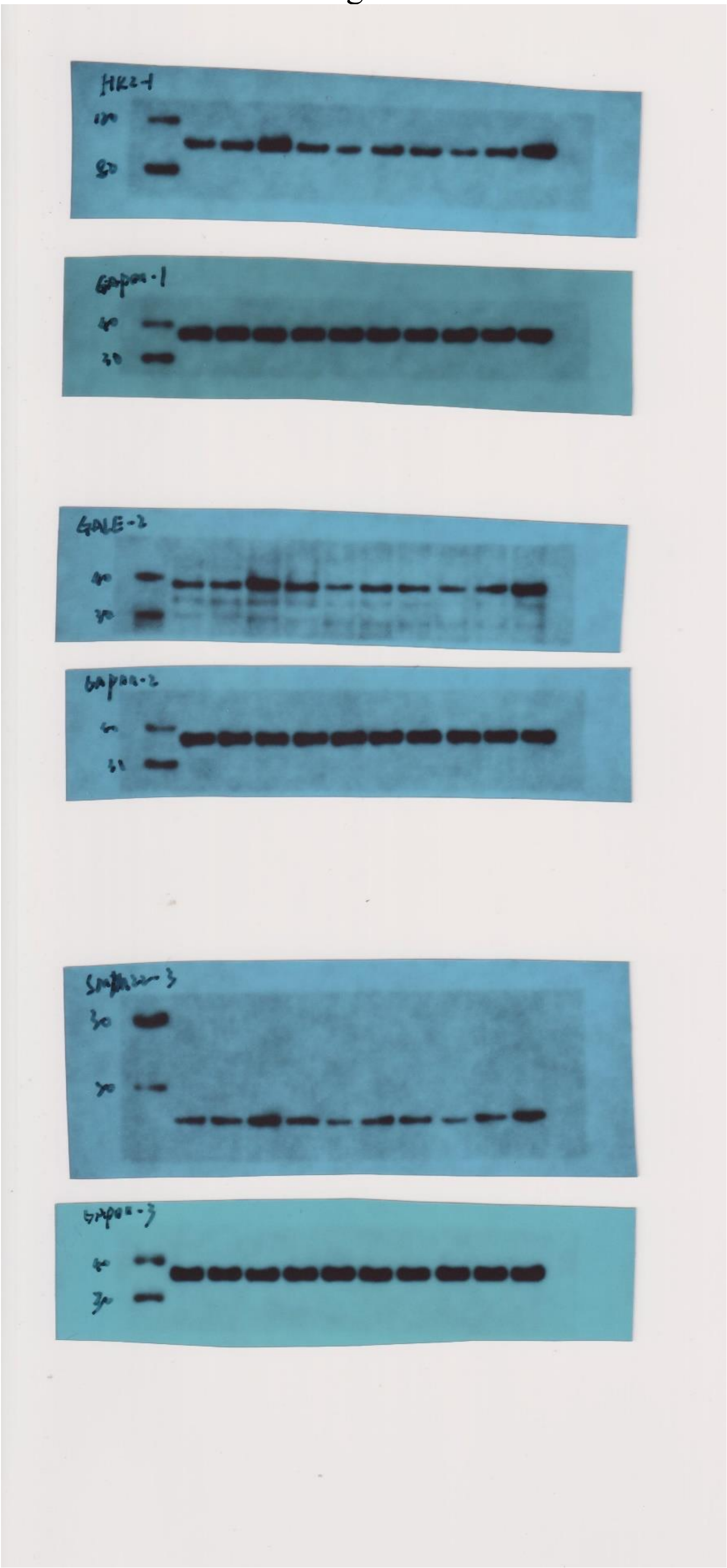

Fig. 6J

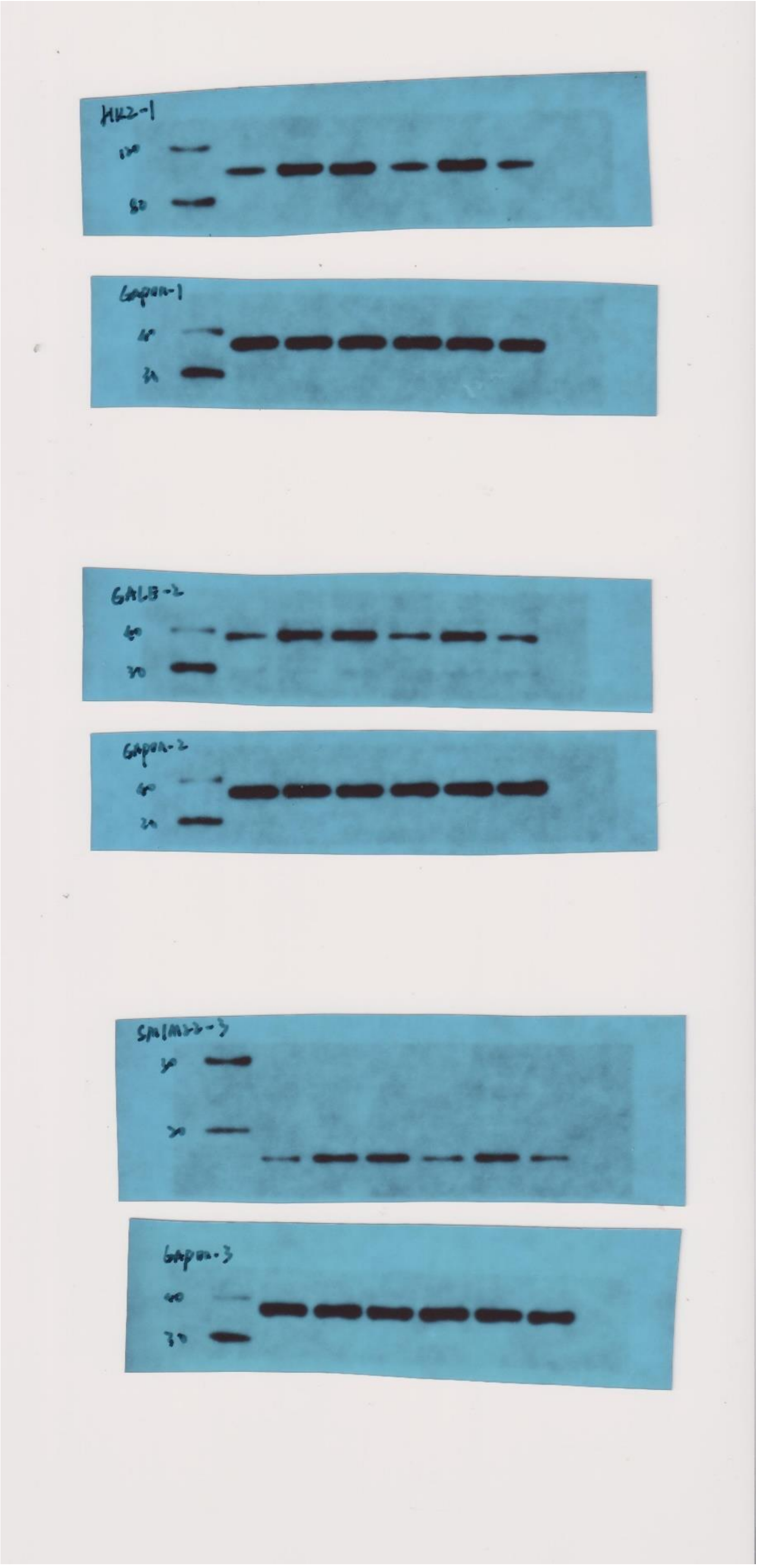

Fig. 6K
